# Supplementary material for: The Evolution and Characterization of the RNA Interference Pathways in Lophotrochozoa
Source: Genome Biol Evol. 2024 May 7;16(5):evae098. doi: 10.1093/gbe/evae098 (PMC11114477; doi:10.1093/gbe/evae098)
Supplement: evae098_Supplementary_Data [file evae098_supplementary_data.zip › Supplementary_materials.zip!/selected_miRNAs/NoGe_selected_miRNAs/mirdeep_pdf_files/NMRB01000161.1_Notospermus_geniculatus_strain_Ushimado_scaffold161,_whole_genome_shotgun_sequence_6107.pdf]

Provisional ID	:	NMRB01000161.1_Notospermus_geniculatus_strain_Ushimado_scaffold161,_whole_genome_shotgun_sequence_6107
Score total	:	4741.1
Score for star read(s)	:	3.9
Score for read counts	:	4732.8
Score for mfe	:	1.3
Score for randfold	:	0
Score for cons. seed	:	3
Total read count	:	9295
Mature read count	:	9089
Loop read count	:	0
Star read count	:	206

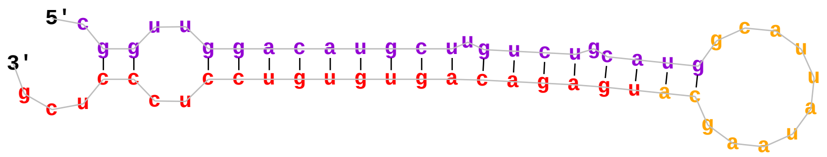

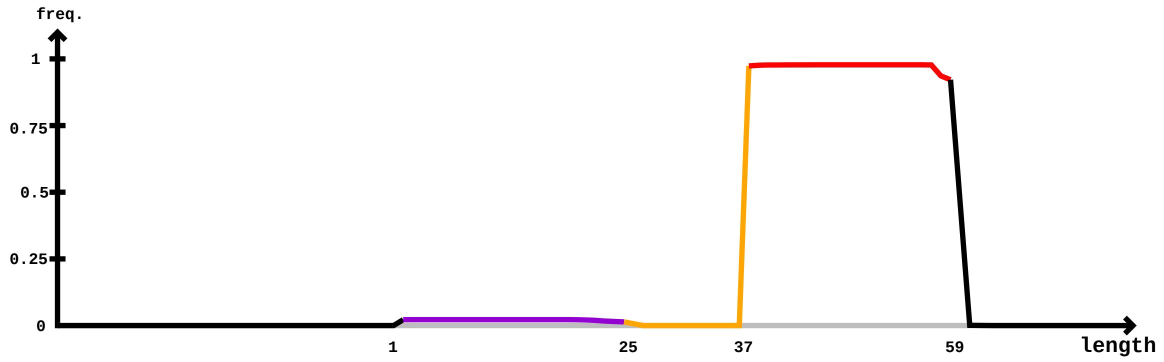

Star Mature

```
5'- ggaauuuuugaucagagaauccaaauuucuuccccgguuggacaugcuugucugcauggcauuauaagcaugagacaguguguccucccucggaaugaaaguuaugucau   -3'    obs
    ggaauuuuugaucagagaauccaaauuucuuccccgguuggacaugcuugucugcauggcauuauaagcaugagacaguguguccucccucggaaugaaaguuaugucau          exp
    (((.(((((.....))))))))...((((((((..((..(((((((((.((((.((((..........)))))))))))))))))..))..)))).))))..........   reads     mm     sample
    ......................................................................ugagacaguguguccucAcucg..................   1         1      Fm2
    ......................................................................ugagacaguguguccucccucU..................   6         1      Fm2
    ......................................................................ugagacaguguguccucUcucg..................   2         1      Fm2
    ......................................................................ugagacaguguguccuAccucg..................   2         1      Fm2
    ......................................................................ugagacaguguguccucccucg..................   120       0      Fm2
    ......................................................................ugagacaguguguccucGcucg..................   2         1      Fm2
    ......................................................................ugagacaguguguccucccucgU.................   35        1      Fm2

    ..................................cgguuggacaugcuuguc..........................................................   2         0      Fm1
    ..................................cgguuggacaugcuugucugcaug....................................................   3         0      Fm1
    ......................................................................ugagacaguguguccucccG....................   1         1      Fm1
    ......................................................................ugagacaguguguccucccu....................   35        0      Fm1
    ......................................................................ugagCcaguguguccucccu....................   1         1      Fm1
    ......................................................................ugagacaguguguccucUcu....................   1         1      Fm1
    ......................................................................ugagacaguguguccucGcu....................   1         1      Fm1
    ......................................................................ugagacaguguguccuUccu....................   1         1      Fm1
    ......................................................................ugagacaguguguccucccuc...................   14        0      Fm1
    ......................................................................ugagacaguguguccucAcuc...................   1         1      Fm1
    ......................................................................ugagacaguguguccuGccuc...................   1         1      Fm1
    ......................................................................ugagacaguguguccucGcucg..................   2         1      Fm1
    ......................................................................ugagacaguguguccucccucC..................   1         1      Fm1
    ......................................................................ugagacaguguguccuccAucg..................   1         1      Fm1
    ......................................................................ugagUcaguguguccucccucg..................   1         1      Fm1
    ......................................................................ugagacaguguguccuUccucg..................   1         1      Fm1
    ......................................................................ugagacaguguguccucAcucg..................   2         1      Fm1
    ......................................................................ugagacaguguguccucccucg..................   282       0      Fm1
    ......................................................................ugagacaguguguccucccucU..................   27        1      Fm1
    ......................................................................ugagacaguguguccucccucA..................   1         1      Fm1
    ......................................................................ugagaUaguguguccucccucg..................   1         1      Fm1
    ......................................................................ugagacaguguguccucccucgU.................   33        1      Fm1
    ......................................................................ugagacaguguguccucccucgA.................   9         1      Fm1
    .......................................................................gagacaguguguccucccucg..................   1         0      Fm1
    .............................................................................guguguccucccucgAaa...............   2         1      Fm1
```

```
                                  Star                                Mature
ggaauuuuugaucagagaauccaaauuucuuccccgguuggacaugcuugucugcauggcauuauaagcaugagacaguguguccucccucggaaugaaaguuaugucau

..................................cgguuggacaugcuugAcugca......................................................    1      1     Mm1
..................................cgguuggacaugcuugucugca......................................................    14     0     Mm1
..................................Ugguuggacaugcuugucugcaug....................................................    1      1     Mm1
..................................cgguuggacaugcuugucugcaug....................................................    17     0     Mm1
...................................Cguuggacaugcuugucugcaug....................................................    1      1     Mm1
......................................................................ugagacaguguguccucccu....................    3      0     Mm1
......................................................................ugagacaguguguccucccuU...................    1      1     Mm1
......................................................................ugagacaguguguccucccuc...................    1      0     Mm1
......................................................................ugagacaguguguccucccucU..................    105    1     Mm1
......................................................................ugagacaguguguAcucccucg..................    1      1     Mm1
......................................................................ugagacNguguguccucccucg..................    1      1     Mm1
......................................................................ugagacUguguguccucccucg..................    2      1     Mm1
......................................................................ugagacaguguguccucAcucg..................    7      1     Mm1
......................................................................ugagacaguguguccuUccucg..................    2      1     Mm1
......................................................................ugagacagugGguccucccucg..................    1      1     Mm1
......................................................................ugagaNaguguguccucccucg..................    2      1     Mm1
......................................................................ugagacaguguguccucccuUg..................    1      1     Mm1
......................................................................Cgagacaguguguccucccucg..................    1      1     Mm1
......................................................................ugagacaguguguccucUcucg..................    12     1     Mm1
......................................................................ugagacaguguguccucGcucg..................    1      1     Mm1
......................................................................ugagacagugAguccucccucg..................    2      1     Mm1
......................................................................ugagacaguAuguccucccucg..................    1      1     Mm1
......................................................................ugagacaguguguccuAccucg..................    5      1     Mm1
......................................................................ugaCacaguguguccucccucg..................    1      1     Mm1
......................................................................ugagacaguguguccuccUucg..................    1      1     Mm1
......................................................................Ngagacaguguguccucccucg..................    2      1     Mm1
......................................................................uAagacaguguguccucccucg..................    1      1     Mm1
......................................................................ugagacaguguguccucccucg..................    1231   0     Mm1
......................................................................ugagacGguguguccucccucg..................    1      1     Mm1
......................................................................ugagacagugAguccucccucgg.................    1      1     Mm1
......................................................................ugagacaguguguccucccucgA.................    6      1     Mm1
......................................................................ugagacaguguguccucccucgg.................    9      0     Mm1
......................................................................ugagacaguguguccucccucgU.................    66     1     Mm1
......................................................................ugagacaguguguccuUccucgg.................    1      1     Mm1

..................................cgguuggacaugcuugucugcau.....................................................    2      0     Mm2
..................................cgguuggacaugcuugucugGau.....................................................    1      1     Mm2
..................................cgguuggacaugcuugucugcaug....................................................    5      0     Mm2
......................................................................ugagacaguguguccuccc.....................    1      0     Mm2
......................................................................ugagacaguguguccucccu....................    10     0     Mm2
......................................................................ugagacaguguguccucccG....................    1      1     Mm2
......................................................................ugagacagugCguccucccu....................    1      1     Mm2
......................................................................ugagacaguguguccucccuU...................    1      1     Mm2
......................................................................ugagacaguguguccucAcuc...................    1      1     Mm2
......................................................................ugagacaguguguccucccuc...................    10     0     Mm2
......................................................................ugagacaguguguccucccucU..................    23     1     Mm2
......................................................................ugGgacaguguguccucccucg..................    1      1     Mm2
......................................................................uAagacaguguguccucccucg..................    1      1     Mm2
......................................................................ugagacaguguguccuccUucg..................    2      1     Mm2
......................................................................ugagacaguguguccucGcucg..................    2      1     Mm2
......................................................................ugagacaguguguccucccucg..................    371    0     Mm2
......................................................................ugagacaguguguccucAcucg..................    5      1     Mm2
......................................................................ugUgacaguguguccucccucg..................    1      1     Mm2
......................................................................ugagacagugAguccucccucg..................    1      1     Mm2
......................................................................ugagacaguguguccucUcucg..................    2      1     Mm2
......................................................................ugagacagugGguccucccucg..................    1      1     Mm2
......................................................................ugaAacaguguguccucccucg..................    1      1     Mm2
......................................................................ugagacaguguguccucccucA..................    2      1     Mm2
......................................................................ugagacaguguguccAcccucg..................    1      1     Mm2
......................................................................ugagacaguguguccuAccucg..................    3      1     Mm2
......................................................................ugagacaguguguccucccucgU.................    138    1     Mm2
......................................................................ugagacaguguguccucccucgA.................    19     1     Mm2
......................................................................ugagacaguguguccucccucgg.................    2      0     Mm2
......................................................................ugagacaguguguccucccucgAaau..............    1      1     Mm2

..................................cgguuggacaugcuugucu.........................................................    9      0     Fm3
..................................cgguuggacaugcuugucug........................................................    13     0     Fm3
..................................Ggguuggacaugcuugucug........................................................    1      1     Fm3
..................................cgguuggacaugcuugucugc.......................................................    13     0     Fm3
```

Star Mature

```
ggaauuuugaucagagaauccaaauuucuuccccgguuggacaugcuugucugcauggcauuauaagcaugagacaguguguccucccucggaaugaaaguuaugucau
.................................cgguuggacaugcuugucugA.......................................................   8     1   Fm3
.................................cgguuggUcaugcuugucugc.......................................................   1     1   Fm3
.................................cgguuggacaugcuuguUugc.......................................................   1     1   Fm3
.................................cgguuggacaugcuugucugcau.....................................................   10    0   Fm3
.................................cgguuggacaugcuuguAugcaug....................................................   1     1   Fm3
.................................cgguuggacaugcuugucugcaug....................................................   88    0   Fm3
.....................................................................ugagacaguguguccuccc.....................   4     0   Fm3
.....................................................................ugagacaguguguccuAccu....................   2     1   Fm3
.....................................................................Ngagacaguguguccucccu....................   1     1   Fm3
.....................................................................ugagacaguguguccucccu....................   281   0   Fm3
.....................................................................ugagacaguguguccucGcu....................   1     1   Fm3
.....................................................................ugagacaguguguccucAcu....................   5     1   Fm3
.....................................................................ugagacaguguguccuUccu....................   1     1   Fm3
.....................................................................Agagacaguguguccucccu....................   1     1   Fm3
.....................................................................ugagacagugAguccucccu....................   1     1   Fm3
.....................................................................ugagacaguguguccucUcu....................   1     1   Fm3
.....................................................................ugagacaguguguccuAccuc...................   1     1   Fm3
.....................................................................ugagacaguguguccucccuc...................   68    0   Fm3
.....................................................................ugagacaguguguccucccuU...................   2     1   Fm3
.....................................................................ugagacaguguguccCcccucg..................   1     1   Fm3
.....................................................................ugagacagugGguccucccucg..................   2     1   Fm3
.....................................................................ugagacaguguguccucccucU..................   182   1   Fm3
.....................................................................ugaUacaguguguccucccucg..................   1     1   Fm3
.....................................................................ugagacaguguguccucccucC..................   1     1   Fm3
.....................................................................ugagaGaguguguccucccucg..................   1     1   Fm3
.....................................................................ugagacaguguguUcucccucg..................   1     1   Fm3
.....................................................................ugagacaguguguccucUcucg..................   17    1   Fm3
.....................................................................ugagacaguguguccuAccucg..................   9     1   Fm3
.....................................................................ugagacagugugCccucccucg..................   1     1   Fm3
.....................................................................ugagacagCguguccucccucg..................   1     1   Fm3
.....................................................................Ngagacaguguguccucccucg..................   2     1   Fm3
.....................................................................ugagacaguguguccucccucA..................   1     1   Fm3
.....................................................................ugagacaguguguccuUccucg..................   5     1   Fm3
.....................................................................ugagacagugCguccucccucg..................   3     1   Fm3
.....................................................................ugagacaguguguAcucccucg..................   2     1   Fm3
.....................................................................ugagacaguguguccucAcucg..................   30    1   Fm3
.....................................................................ugagaUaguguguccucccucg..................   1     1   Fm3
.....................................................................ugagacaguguguccucccucg..................   3239  0   Fm3
.....................................................................Agagacaguguguccucccucg..................   2     1   Fm3
.....................................................................uUagacaguguguccucccucg..................   2     1   Fm3
.....................................................................ugagacaguguguccuccAucg..................   2     1   Fm3
.....................................................................ugCgacaguguguccucccucg..................   1     1   Fm3
.....................................................................ugagacaguguguccAcccucg..................   1     1   Fm3
.....................................................................ugagacagugAguccucccucg..................   6     1   Fm3
.....................................................................ugagacUguguguccucccucg..................   2     1   Fm3
.....................................................................ugagacaguguguccuGccucg..................   3     1   Fm3
.....................................................................ugagacaguguguccucccuUg..................   2     1   Fm3
.....................................................................ugagacaguguguccucGcucg..................   16    1   Fm3
.....................................................................ugGgacaguguguccucccucg..................   2     1   Fm3
.....................................................................ugagacaguguguccucccucgU.................   1240  1   Fm3
.....................................................................ugagacaguguguccucccucgg.................   28    0   Fm3
.....................................................................ugagacaguguguccucccucgA.................   214   1   Fm3
.....................................................................ugagacaguguguccucccucUg.................   1     1   Fm3
......................................................................gagacaguguguccucccu....................   1     0   Fm3
......................................................................gagacaguguguccucccucg..................   8     0   Fm3
......................................................................gagacaguguguccucccucgU.................   11    1   Fm3
.......................................................................agacaguguguccucccucg..................   10    0   Fm3
.........................................................................acaguguguccucccucg..................   4     0   Fm3
..........................................................................caguguguccucccucgU.................   1     1   Fm3

.................................cgguuggacaugcuugucugc.......................................................   1     0   Mm3
.................................cgguuggacaugcuugucugca......................................................   1     0   Mm3
.................................cgguuggacaugcuugucugcaug....................................................   12    0   Mm3
....................................................................augagacaguguguccucccuU...................   1     1   Mm3
.....................................................................ugagacaguguguccuccc.....................   1     0   Mm3
.....................................................................ugagacaguguguccucccu....................   31    0   Mm3
.....................................................................ugagacaguguguccucUcu....................   1     1   Mm3
.....................................................................ugagacaguguguccuGccuc...................   1     1   Mm3
.....................................................................ugagacaguguguccucccuc...................   27    0   Mm3
.....................................................................ugagacaguguguccucccuU...................   4     1   Mm3
```

```
                                  Star                               Mature
ggaauuuuugaucagagaauccaaauuucuuccccgguuggacaugcuugucugcauggcauuauaagcaugagacaguguguccucccucggaaugaaaguuaugucau
......................................................................uAagacaguguguccucccucg..................   1     1    Mm3
......................................................................ugagacaguguguccucccucA..................   5     1    Mm3
......................................................................ugagacagugugCccucccucg..................   1     1    Mm3
......................................................................ugagacaguguguccucAcucg..................   4     1    Mm3
......................................................................ugagacagugGguccucccucg..................   2     1    Mm3
......................................................................ugagacaguguguccuUccucg..................   1     1    Mm3
......................................................................ugagacaguguguccucccucU..................   68    1    Mm3
......................................................................ugagacaguguguccucUcucg..................   6     1    Mm3
......................................................................ugagacagugCguccucccucg..................   1     1    Mm3
......................................................................ugagacaguguguccuAccucg..................   3     1    Mm3
......................................................................ugagacaguguguccucccucg..................   515   0    Mm3
......................................................................ugagacaguguguccuGccucg..................   1     1    Mm3
......................................................................ugagacaguguguccucGcucg..................   2     1    Mm3
......................................................................ugagacaguguguccucccucgU.................   292   1    Mm3
......................................................................ugagacaguguguccucccucgg.................   4     0    Mm3
......................................................................ugagacaguguguccucccucgA.................   26    1    Mm3
......................................................................ugagacaguguguccucccucgUa................   3     1    Mm3
......................................................................ugagacaguguguccucccucgUaa...............   1     1    Mm3
......................................................................ugagacaguguguccucccucgAaa...............   1     1    Mm3
.......................................................................gagacaguguguccucccucgU.................   1     1    Mm3
```
